# Lethality of Sesquiterpenes Reprogramming Red Palm Weevil Detoxification Mechanism for Natural Novel Biopesticide Development

**DOI:** 10.3390/molecules24091648

**Published:** 2019-04-26

**Authors:** Abid Hussain, Muhammad Rizwan-ul-haq, Ahmed Mohammed AlJabr, Hassan Al-Ayedh

**Affiliations:** 1 Laboratory of Bio-Control and Molecular Biology, Department of Arid Land Agriculture, College of Agricultural and Food Sciences, King Faisal University, Hofuf 31982, Saudi Arabia; solvia_aah@yahoo.com (A.H.); mianrizwan15@gmail.com (M.R.-u.-h.); 2 National Agriculture Technology Center, Life Science & Environment Research Institute, King Abdulaziz City for Science & Technology, P.O. Box 6086, Riyadh 11442, Saudi Arabia; alayedh@kacst.edu.sa; 3 Ministry of Environment, Water and Agriculture, Riyadh 11442, Saudi Arabia; 4 RPW Consultant, United Nations, Food and Agriculture Organization (FAO), Riyadh 11442, Saudi Arabia

**Keywords:** sesquiterpenes, plant secondary metabolites, *Rhynchophorus ferrugineus*, biopesticides, natural products, host defense, detoxification mechanism

## Abstract

Natural biopesticide development for invasive populations of red palm weevils is mainly responsible for the destruction of date palms and demands an extensive screening program of plant secondary metabolites. In the current study, the pesticidal potential of sesquiterpenes (C_15_ H_24_), an important class of plant secondary metabolites primarily composed of three isoprene units, was evaluated by laboratory toxicity, feeding performance bioassays, and host detoxification gene expression patterns. Dose-mortality response bioassays performed against mid-aged eighth-instar red palm weevil larvae revealed dose-dependent mortality. Only three sesquiterpenes, including Farnesol (LD_50_ = 6559 ppm) and Farnesyl acetate (LD_50_ = 7867 ppm), are considered to have significant toxicity, with Picrotoxin (LD_50_ = 317 ppm) being the most toxic. Furthermore, highly toxic sesquiterpene (Picrotoxin) established in the current study tremendously reduced the feeding performance indices, including the efficacy of conversion of digested food (ECD) (81.74%) and the efficacy of conversion of ingested food (ECI) (73.62%). The least toxic sesquiterpenes, including β-Caryophyllene, (+)-Cedrol, Nerolidol, (+)-Nootkatone, and Parthenolide, observed in the current study failed to impart significant reductions of ECI and ECD indices. Lethality of the least toxic sesquiterpenes was overcome by greatly inducing gene expressions of *Glutathione S transferase (GST)* and *Cytochrome P450*. These encouraging results enabled us to suggest Picrotoxin as a promising biopesticide for the control of red palm weevil infestations.

## 1. Introduction

Red palm weevil, *Rhynchophorus ferrugineus* (Olivier) (Coleoptera, Curculionidae), is considered the most devastating invasive pest and is mainly responsible for the destruction of date palms in the Arabian Peninsula. The cryptic lifestyle of red palm weevil grubs in the trunk exhibits serious management challenges. Red palm weevil control mainly depends on the intensive use of synthetic pesticides as a soil treatment, tree fumigation, trunk injection, wound dressing, and crown drenching of infested palms [1]. However, the indiscriminate use of synthetic pesticides is mainly responsible for pesticide residues in dates [2], environmental pollution [3], resistance development [4], and detrimental effects on wildlife [5]. Therefore, the use of pesticides is discouraged, and the concept of eco-friendly natural pesticide development is gaining attention and popularity.

Plant secondary metabolites are an untapped reservoir of chemicals with numerous potential uses [6,7]. They have expressed deleterious effects, including (1) reduced fecundity, (2) molt-inhibitory, (3) toxic, (4) respiratory inhibition, (5) growth retarding, (6) oviposition deterrence, (7) suppression of calling behavior, and (8) repellency against target pest species [8,9,10,11]. The incorporation of plant secondary metabolites into the Integrated Pest Management strategy is advantageous due to their broad spectrum of activity [12], and the fact that they pose the least risk to the environment [7] and biodegradability [12]. In addition, sesquiterpenes trigger a host defense mechanism to reduce pest damage [13].

Using plant secondary metabolites in controlling red palm weevils was neglected in the past. Therefore, little data are available on this aspect. A great deal of focus remained on the insecticidal potential of crude plant extracts against red palm weevils [14,15,16,17]. Little focus remained on the insecticidal potential of plant secondary metabolites, including rotenone, limonine, coumarin, and piperine, against red palm weevils [18,19,20]. A recent study screened the insecticidal potential of a wide range of acyclic monoterpenes against red palm weevil larvae [20]. Their results revealed insecticidal and growth inhibitory potential of Coumarin against red palm weevil larvae [20,21]. Insecticidal potential of sesquiterpenes (an important class of terpenes comprising three isoprenes) is well known against other pest species, including *Aedes aegypti*, *Coptotermes formosanus*, *Dermacentor variabilis, Musca domestica, Nilaparvata lugens*, *Pediculus capitis*, *Periplaneta americana*, *Sitophilus zeamais, S. oryzae*, *Stephanitis pyrioides*, and *Tribolium castaneum* [22,23,24,25,26,27,28]. Surprisingly, there has not been a single study on the insecticidal potential of sesquiterpenes against red palm weevils. The current study was designed to explore, for the first time, the pesticidal potential of red palm weevil larvae by (1) screening the most potent sesquiterpene among β-Caryophyllene, (+)-Cedrol, Farnesol, Farnesyl acetate, Nerolidol, (+)-Nootkatone, Parthenolide, and Picrotoxin by dose-mortality response laboratory bioassays; (2) evaluating the impacts of selected sesquiterpenes on the growth and development of red palm weevil larvae by dietary feeding performance bioassays; (3) exploring the impact of the red palm weevil detoxification mechanism against sesquiterpenes by quantifying detoxification-related genes including *Glutathione S transferase (GST)*, *Cytochrome P450*, and *Esterase* by quantitative real-time PCR (qRT-PCR) in order to pave the way for safer natural biopesticides development against red palm weevil infestations.

## 2. Results

### 2.1. Toxicity of Sesquiterpenes against Red Palm Weevil Larvae

**Figure 1:**
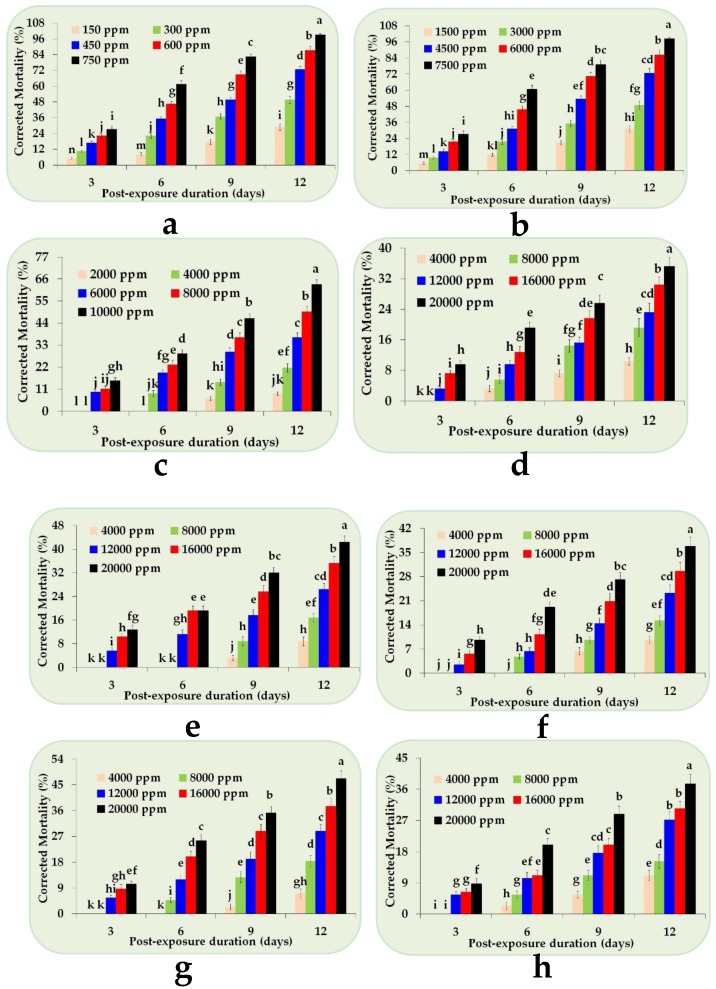
Corrected cumulative (%) dose mortality response of red palm weevil larvae against (**a**) Picrotoxin; (**b**) Farnesol; (**c**) Farnesyl acetate; (**d**) β-Caryophyllene; (**e**) (+)-Cedrol; (**f**) Nerolidol; (**g**) (+)-Nootkatone; and (**h**) Parthenolide. Each bar (mean ± SE) is the mean of five replicates. The bars followed by different letter(s) are significantly different (Fisher’s Least Significant Difference test, α *=* 0.05).

The insecticidal potential of all tested sesquiterpenes against red palm weevil larvae, evaluated here by diet-incorporated dose-mortality response bioassays, exhibited dose-dependent mortality response, as shown in Figure 1a–h. In the current study, Picrotoxin (Figure 1a) was found to be the most toxic compound against red palm weevil larvae resulting in the least LD_50_ value of 317 ppm (Table 1). Feeding the eighth-instar red palm weevil larvae a diet incorporated with different doses of Picrotoxin imparted significant mortality at all studied time intervals (*F* = 126.05; *df* = 3, 64; *p* < 0.0001) with different doses (*F* = 1730.14; *df* = 4, 64; *p* < 0.0001) and their interaction (*F* = 20.52; *df* = 12, 64; *p* < 0.0001).

Similarly, Farnesyl acetate also revealed significant differences in mortality at all the tested doses (*F* = 1262.73; *df* = 4, 64; *p* < 0.0001) at different time intervals (*F* = 95.39; *df* = 3, 64; *p* < 0.0001) and their interaction (*F* = 15.99; *df* = 12, 64; *p* < 0.0001), as shown in Figure 1c. In contrast, LD_50_ values for the compounds such as β-Caryophyllene, (+)-Cedrol, Nerolidol, (+)-Nootkatone, and Parthenolide could not be calculated, because these sesquiterpenes failed to impart 50% mortality even at the highest dose (20000 ppm) during the course of experimentation (Table 1). Despite this, the interaction of the doses of these least potent sesquiterpenes at different time intervals revealed significant differences in mortality for β-Caryophyllene (*F* = 5.24; *df* = 12, 64; *p* < 0.0001), (+)-Cedrol (*F* = 14.02; *df* = 12, 64; *p* < 0.0001), Nerolidol (*F* = 5.45; *df* = 12, 64; *p* < 0.0001), (+)-Nootkatone (*F* = 11.99; *df* = 12, 64; *p* < 0.0001), and Parthenolide (*F* = 4.12; *df* = 12, 64; *p* < 0.0001), as shown in Figure 1d–h.

### 2.2. Growth Retarding Activities of Sesquiterpens

Red palm weevil eighth-instar larvae fed on a diet incorporated with sesquiterpenes revealed significant variations in their feeding indices, including efficacy of conversion of ingested food (ECI), efficacy of conversion of digested food (ECD), and approximate digestibility (AD). We recorded the highest reduction in ECD (81.74%) from red palm weevil larvae fed on a diet incorporated with the most toxic, Picrotoxin. Furthermore, we recorded significant variations among all treatments (*F* = 118; *df* = 8, 36; *p* < 0.0001). However, the lowest reduction (<11%) was observed from the diets incorporated with β-Caryophyllene (6.95%), (+)-Cedrol (10.32%), Nerolidol (7.29%), and Parthenolide (4.53%), as shown in Table 2.

Picrotoxin, established in the current study as the most potent compound, tremendously reduced the ECI (73.62%) of red palm weevil larvae (Table 2). The sesquiterpenes including β-Caryophyllene (2.81%), (+)-Cedrol (4.27%), Nerolidol (3.17%), (+)-Nootkatone (7.23%), and Parthenolide (2.45%) failed to impart tremendous reduction of ECI compared to the control treatment diet (Table 2). Overall, all the treatments showed significant variations (*F* = 144; *df* = 8, 36; *p* < 0.0001). Furthermore, the ECI index of β-Caryophyllene, (+)-Cedrol, Nerolidol, and Parthenolide remained statistically at the same level of significance (Table 2). Overall, we recorded the directly proportional relationship of toxicity of the sesquiterpenes with growth indices (ECI and ECD). Potent sesquiterpenes revealed the highest reduction of ECI and ECD. 

To the contrary, we recorded an inversely proportional relationship between AD and toxicity (Table 2). Picrotoxin, the most potent reported here, significantly enhanced (30.80%) red palm weevil larval AD compared with the control. In addition, AD significantly increased upon the exposure to different sesquiterpenes (*F*= 238; *df* = 8, 36; *p* < 0.0001).

### 2.3. Regulation of Detoxification Genes of Red Palm Weevils in Response to Sesquiterpenes

#### 2.3.1. *Cytochrome P450* Gene Expression of Red Palm Weevil Larvae

Toxicity of tested sesquiterpenes induced different levels of the *Cytochrome P450* gene (Table 3). The quantitative expression of *Cytochrome P450* revealed significant differences (*F* = 197; *df* = 7, 32; *p* < 0.0001). Larvae fed on a diet incorporated with Picrotoxin and Farnesol tremendously reduced the expression of tested detoxification genes such as *Cytochrome P450*. Among all the sesquiterpenes, the least toxic Parthenolide greatly enhanced *P450* expression of red palm weevil larvae and remained significant at the highest level. However, β-Caryophyllene and (+)-Cedrol also greatly enhanced the expression of *P450* genes and remained significant at the same level of significance (Table 3).

#### 2.3.2. *Glutathione S-Transferase* Gene Expression of Red Palm Weevil Larvae

Artificial diet incorporated with different sesquiterpenes induced different levels of *GST* expressions, resulting in significant differences (*F* = 266; *df* = 7, 32; *p* < 0.0001) in their quantitative expressions. The least toxic sesquiterpenes established in the current study against red palm weevil larvae, such as β-Caryophyllene, (+)-Cedrol, Nerolidol, (+)-Nootkatone, and Parthenolide, showed higher expressions of *GST* (>15%) compared with potent sesquiterpenes. Among all the treatments, Picrotoxin failed to induce *GST* expression of red palm weevil larvae and remained significant at the lowest level (Table 3).

#### 2.3.3. *Esterase* Gene Expression of Red Palm Weevil Larvae

Quantitative expression of the detoxification gene, *Esterase*, also showed significant differences in the expression (*F* = 45; *df* = 7, 32; *p* < 0.0001) from the mid-gut of red palm weevil larvae. Overall, all the sesquiterpenes failed to induce higher expressions of *Esterase* among red palm weevil larvae (Table 3).

## 3. Discussion

Development of insecticide resistance demands the exploration of newer, plant-based, eco-friendly biopesticides for controlling the infestations of red palm weevils. Our drafted screening program to find the most toxic sesquiterpenes reported here—for the first time—the toxicity of Picrotoxin against red palm weevils. The most potent sesquiterpene, Picrotoxin, established in the current study tremendously disturbed the larval growth and evaded the host detoxification mechanism. The red palm weevil detoxification mechanism displayed a massive and rapid reprogramming of gene expressions in response to the least toxic sesquiterpenes for their survival.

Higher larval mortality of red palm weevils was recorded from the artificial diet incorporated with Picrotoxin. The exposed red palm weevil larvae failed to tolerate Picrotoxin. Furthermore, Picrotoxin-fed larvae became sluggish, which ultimately led toward the death of > 96% of the larvae during the course of experimentation. Picrotoxin, a known natural plant toxin isolated from flowing plants (Menispermaceae), is the representative of non-competitive antagonists (NCAs) [29]. In addition to use as ectoparasiticides and insecticides, they also play very important roles as molecular tools in the studies of γ-aminobutyrate (GABA) receptors at physiological and biochemical levels [30]. Therefore, Picrotoxin as GABAergic receptors, which act by modulating and blocking chlorine channels [31,32], are listed as antidotes against barbiturate poisoning [33], convulsants [34], memory deficiency in animals [32], and they can speed the mammalian circadian clock [35]. In insects, Picrotoxin tested against many insect species showed various activities, such as acting as a pesticide [36,37], acting as a modulator [38], abolishing the oscillatory synchronization [39], and causing a loss in the ability to perceive odor mixtures of configural stimulants [40]. In the past, another neurotoxic toxic compound, Fipronil, with the antagonism of GABA as the mechanism of toxicity was tested against red palm weevils as quarantine treatment [41], Endotherapic injection [42], and laboratory toxicity bioassays [43]. However, Picrotoxin (LD_50_ of 317 ppm) in the current study was found to be the most potent compound against red palm weevil larvae compared with other neurotoxins, including Fipronil, which showed 773.78 ppm of red palm weevil larval LC_50_ [43]. The toxicity of this compound coincides with AlJabr et al., who reported the toxicity (LD_50_ of 0.672 g/L) of the plant secondary metabolite, Coumarin, against red palm weevil larvae [20]. In the past, the toxicity of Picrotoxin against red palm weevils had never been tested. This is the first study evaluating and reporting the toxicity of Picrotoxin against red palm weevil larvae. These findings enable us to suggest Picrotoxin as a great alternate option for red palm weevil management.

In the current study, similarly high ECI (73.62%) and ECD (81.74%) reductions were calculated among larvae fed on a diet incorporated with Picrotoxin. The reduced indices of ECI and ECD in response to the most potent Picrotoxin might have been because of the shortfall in food reserves, which might have appeared because of (1) the intake of less food due to toxicity, and (2) the utilization of most of the energy for the host defense in place of the host growth and development. The previous research findings also claimed reductions in ECI and ECD in response to toxins, suspensions, and plant extracts. In this regard, Hussain et al. [44] reported tremendous reduction in ECI and ECD indices of *Ocinara varians* Walker larvae against suspensions of entomopathogenic fungi. The findings of Koul et al. [45] reported the biological activity of limonoids against *Helicoverpa armigera* (Hubner) and *Spodoptera litura* (Fabricius). Their results showed larval growth inhibition in response to three tested limonoids. However, they suggested that growth inhibition might have been due to feeding deterrence. Our results are in line with Silva et al. [46], who reported similar growth patterns of *Anagasta kuehniella* (Zeller) larvae fed on an artificial diet incorporated with the extract of *Croton urucurana*. The findings of [21] also reported a tremendous reduction in ECI and ECD indices of red palm weevil larvae fed on a diet incorporated with a plant secondary metabolite. They suggested that the reduced growth in the form of ECI and ECD indices might have been due to the utilization of most of the energy reserves to combat the host defense instead of being used for growth and development. Such energy-deficient larvae after slow growth ultimately led to death [20,21]. Another study conducted on the toxicity of insecticides against red palm weevil larvae also revealed similar growth patterns from the most toxic insecticides [4]. In addition to Picrotoxin, Farnesol (LD_50_ of 6559 ppm) also showed favorable results in terms of toxicity. Similar to Picrotoxin, Farnesol also tremendously reduced ECD (39.69%) and ECI (26.91%) indices of red palm weevil larvae. Such reductions in ECI and ECD indices are in agreement with the previous study on the susceptibility exploration of red palm weevils towards fungal infections [47]. They reported the highest reductions in ECI and ECD indices upon infections with the most pathogenic isolates of entomopathogenic fungi. Their findings suggested that pathogen virulence regulated the growth and defense mechanism of red palm weevils. Furthermore, they suggested that the least virulent isolate of *Isaria fumosorosea* 03011 failed to impart significant reductions, as depicted in the current study in the case of the least toxic sesquiterpenes, including β-Caryophyllene, (+)-Cedrol, Nerolidol, and Parthenolide. Similarly, conidial suspension of *Beauveria Bassiana* isolate B8465 also revealed low inhibition of ECI and ECD indices of red palm weevil different instar (fourth, eighth, and twelfth) larvae [48]. They suggested that entomopathogenic fungal isolate lacking a right set of virulence traits could not impart pathogenesis in the target host, resulting in the failure to impart growth reductions. Our results are also in line with AlJabr et al. [20], who reported the similar negligible response of plant secondary metabolites such as methyl isoeugenol and methyl eugenol on the growth indices of red palm weevil larvae. Based on these findings, we might suggest that a reduction in feeding performance indices (ECI and ECD) is directly proportional to the toxicity of sesquiterpenes. Such a situation might arise because of the shortfall of energy reserves. These energy-deficient larvae utilize most of their energy reserves in physiological activities. Under such circumstances, exposed larvae grow very slowly, which ultimately leads towards death.

AD is also an important feeding performance index recorded in the current study. Feeding performance bioassays revealed a 30.80% increase in the AD of Picrotoxin compared with the control treatment. A similar increase in AD value is in line with the findings of AlJar et al. [20], who reported that the toxic, Coumarin, significantly enhanced the digestibility of energy-deficient larvae of red palm weevils in order to meet their energy demands. Enhanced AD response has also been reported among red palm weevil larvae to fungal suspensions [48], plant secondary metabolites [21,49], and plant extracts [18], as well as *Ocinara varians* Walker larvae infected with conidial suspensions of entomopathogenic fungi [44,50]. A similarly enhanced response of AD in response to labramin among *A. kuehniella* was reported by Martinez et al. [51]. On the other hand, the least toxic compound, Parthenolide, revealed in the current study that it remained statistically near the control treatment. In the past, a similar response of AD from non-toxic treatments was highlighted by AlJabr et al. [20]. They reported Methyl eugenol as the least toxic compound that failed to regulate abnormal growth of red palm weevil larvae. In contrast, a potent treatment, especially Coumarin, greatly affected growth, resulting in a significant increase in the AD of exposed larvae, which is in agreement with the current study in the case of Picrotoxin. Our results are also in line with the previous study on the insecticidal and growth retarding potential of black pepper extracts and their constituent, Piperine, against red palm weevil larvae [18]. Results revealed the highest AD index from the most potent Piperine-fed larvae. Such a tremendous increase in AD reported here and claimed before is mainly because of the fact that exposed larvae demand extra energy to regulate the host defense mechanism. In order to meet emerging energy demands, exposed larvae utilized their intrinsic capabilities, which ultimately enhanced the AD of their limited foodstuff.

Toxicity of the compounds expedites the regulation of detoxification-related genes by increasing the host metabolism. In this regard, host defense-related genes, especially *Glutathione S Transferase*, *Cytochrome P 450*, and *Esterase*, are known for their importance in host detoxification mechanisms. Previous studies revealed the increase in *GST* expression in resistant red palm weevil populations provided with a cypermethrin-incorporated artificial diet [4]. In the current study, we quantified the expression patterns of *GST*, *Cytochrome P450*, and *Esterase*. Results revealed tremendous expression of all tested detoxification-related genes in response to the least potent compounds, including β-Caryophyllene, (+)-Cedrol, Nerolidol, (+)-Nootkatone, and Parthenolide. Among all the tested detoxification genes, *GST* showed the highest response against the least toxic compounds. The enhanced *GST* response of red palm weevils fed on a diet incorporated with less toxic compounds corroborates a recently published study on the modulation of the host detoxification system in response to an important plant secondary metabolite [20]. Our results are also in line with the previous enhanced response of toxin-pathogen application against date palm dust mites [49]. Furthermore, their study suggested that host detoxification genes such as *GST*, *Cytochrome P450*, and *Esterase* detoxify the toxin by increasing their solubility, which leads towards the toxin degradation and removal from the host body. The enhanced detoxification genes expression patterns greatly reduced the lethality of the tested compound. These findings enable us to suggest that Picrotoxin, the most potent compound established in the current study, disguised the expressions of detoxification genes, which ultimately imparted the least LD_50_ value (317 ppm). However, expression of *Esterase*, which we found very little of in the current study, does not mean that they are not engaged in the detoxification mechanism. We might suggest that low expression of the *Esterase* gene might have been due to low specificity with the tested sesquiterpenes. These encouraging results pave the way towards the development of eco-friendly red palm weevil management option.

## 4. Materials and Methods

### 4.1. Insects Rearing

The population of red palm weevil was obtained from an already developed culture maintained under controlled conditions in the growth chamber at 30 ± 1ºC, 75% ± 5% relative humidity (RH) at the Laboratory of Bio-control and Molecular Biology, King Faisal University, Saudi Arabia. Third-instar red palm weevil larvae were reared on an artificial diet standardized in our previous study [48].

### 4.2. Sesquiterpenes

**Figure 2:**
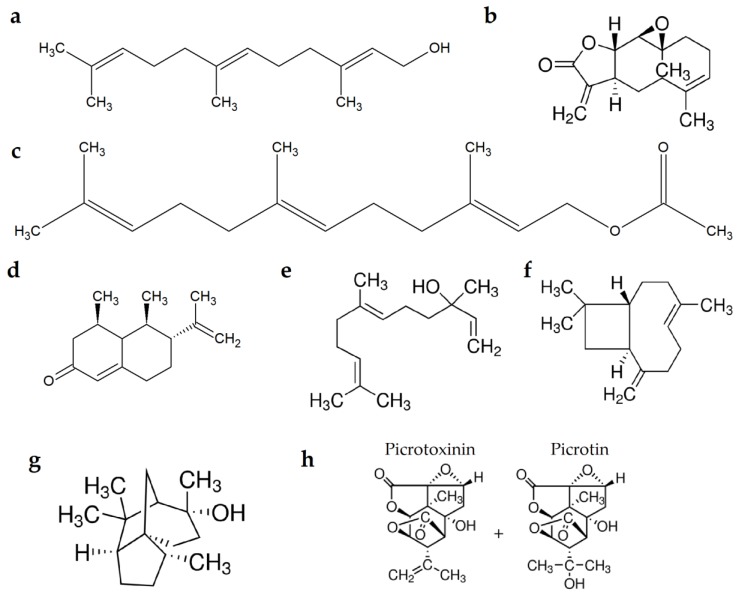
Structures of studied sesquiterpenes, (**a**) Farnesol, (**b**) Parthenolide, (**c**) Farnesyl acetate, (**d**) (+)-Nootkatone, (**e**) Nerolidol, (**f**) β-Caryophyllene, (**g**) (+)-Cedrol, and (**h**) Picrotoxin.

Eight sesquiterpenes including β-Caryophyllene (Cat # W225207), (+)-Cedrol (Cat # W521418-1KG-K), Farnesol (Cat # F203-100G), Farnesyl acetate (Cat # 45895-10ML-F), Nerolidol (Cat # W277207-1KG-K), (+)-Nootkatone (Cat # 74437-2.5G), Parthenolide (Cat # P0667-25MG), and Picrotoxin (Cat # P1675-5G), as shown in Figure 2, were purchased from Sigma Aldrich, UK.

### 4.3. Laboratory Toxicity Testing of Sesquiterpenes against Red Palm Weevil Larvae

The sesquiterpenes selected for the current study were never previously tested against red palm weevils. Therefore, preliminary range-finding dose-mortality bioassays were conducted to finalize the doses for all tested sesquiterpenes. The finalized five doses of each tested sesquiterpene including β-Caryophyllene, (+)-Cedrol; Farnesol, Farnesyl acetate; Nerolidol, (+)-Nootkatone; Parthenolide, and Picrotoxin were prepared by dissolving each tested sesquiterpene in 0.05% of the appropriate solvent. All the components of the artificial diet disclosed in our previous studies were mixed in 500 mL of each tested sesquiterpene at the required dose [18,20]. The control treatment diets were prepared using the respective solvent at a dose of 0.05%. Twenty-five mid-aged (eighth-instar) red palm weevil larvae were singly-fed on the treated artificial diet for 12 days in perforated plastic bowls. Overall, five replicates, each from a different generation, were prepared. Bioassays were repeated over time. Each experimental unit was kept in the growth chamber at 30 ± 1 °C, 75% ± 5% RH. Mortality data were recorded after 24 h feeding on the diets. Control mortality data were subtracted from the treatment mortality data using Abbott’s formula [52].

$$\mathrm{Coreected}\;\%\;\mathrm{Mortality}=\left(1-\frac{n\;\mathrm{in}\;T\;\mathrm{after}\;\mathrm{treatment}}{n\;\mathrm{in}\;\mathrm{Co}\;\mathrm{after}\;\mathrm{treatment}}\right)*100$$

where n stands for “insect population”; T stands for “treated”; and Co stands for “control”.

Cumulative corrected larval mortality data were angularly transformed for data homogenization. Homogenized cumulative corrected mortality data were analyzed by repeated measures ANOVA at different time intervals with Fisher’s LSD test [53]. Probit analysis was performed to determine the lethal dose (LD_50_) of sesquiterpenes showing 50% red palm weevil larval mortality.

### 4.4. Larval Growth and Development Retarding Abilities of Tested Sesquiterpenes

Eighth-instar red palm weevils were used to evaluate the impact of sesquiterpenes on the larval feeding performance. The larvae were provided with an artificial diet incorporated with either sesquiterpenes β-Caryophyllene, (+)-Cedrol, Farnesol, Farnesyl acetate, Nerolidol, (+)-Nootkatone, and Parthenolide at a single dose of 317 ppm. The dose 317 ppm was the LD_50_ of the most potent sesquiterpene (Picrotoxin). A measured amount of the artificial diet incorporated with each tested sesquiterpene was separately provided in perforated plastic bowls. Each treatment comprised 25 singly-fed eighth-instar red palm weevil larvae. Five replicates were prepared similarly using different generations of red palm weevils. Each experimental unit was kept for 72 h at 30 ± 1 °C, 75% ± 5% RH. After 72 h post feeding, ECD, ECI, and AD indices were calculated on the basis of dry frass weight, food consumed, and weight gained basis, as described in previous studies [47,48,50]. Variations in growth indices as a result of feeding on the diet incorporated with different sesquiterpenes were calculated by one-way ANOVA and Fisher’s LSD test using Statistical Analysis Software (SAS) [54].

### 4.5. Physiological Impacts of Sesquiterpenes on the Host Detoxification Defense Mechanism

The mid-gut portion of the alimentary canal of eighth-instar red palm weevil larvae was used to determine the expression patterns of detoxification genes, including *GST*, *Esterase*, and *Cytochrome P450*. Each treatment (sesquiterpenes) at a single dose of 317 ppm was incorporated in the artificial diet to feed eighth-instar red palm weevil larvae for 72 h in the incubator under controlled conditions. Five replicates were prepared by five different larval gut samples. After dissection in saline, the mid-gut portion of each larva was ground in liquid nitrogen for total RNA extraction using RNeasy^®^ Universal Mini Kit (Qiagen Cat No 73404) following manufacturer recommendations. Each extracted total RNA was used to synthesize first strand cDNA using a commercially available kit (Clontech Cat # 6110A). The synthesized first strand cDNA product of each reaction was stored at −20 °C. The primers for *GST* (F 5′-ATAGCCAACCACCACTGTCG-3′ and R 5′-CGTTCCTCTTGCCGCTAGTT-3′) with accession number KR902496; *Esterase* (F 5′-ACCTACAAGAATCCGACGCC-3′ and R 5′-ACTCCGAAACTTTGGGCCAT-3′) with accession number KT748822; *Cytochrome P450* (F 5′-TGGAGAAACACCCGCAAGAA-3′ and R 5′-CGGCGATTTTGCCTACCAAG-3′) with accession number KT748789; and β-Actin (F 5′-AAAGGTTCCGTTGCCCTGAA-3′ and R 5′-TGGCGTACAAGTCCTTCCTG-3′) with accession number KM438516, designed using the online tool on NCBI, were purchased from Macrogen, Korea (http://www.macrogen.com/en/company/summary.php). β-Actin was used as an internal control. Differential expressions of *GST*, *Esterase*, and *Cytochrome P450* were determined using SYBR^®^ Premix Ex Taq™ II kit (TaKaRa Clontech, France) in CFX96 Touch^TM^ (Bio-Rad, UK) according to the manufacturer’s protocol. Numerical values obtained from each experimental unit were compared with those of the control by relative fold expression obtained by transforming the obtained results into absolute values using 2^−ΔΔCt^ [55]. The relative expression of each gene was set to 1 for the uninfected (control) treatment. SAS Institute (2000) was used to determine variations among treatments by ANOVA and Fisher’s LSD test.

## 5. Conclusions

In conclusion, our results demonstrated Picrotoxin as the most potent sesquiterpene against red palm weevils. An artificial diet incorporated with Picrotoxin was proven to inhibit the growth activities and to reprogram the detoxification defense mechanism of red palm weevils. Picrotoxin, a natural plant neurotoxin, is an important addition in the list of red palm weevil controlling products. Future research should focus on various aspects including formulation, safety, and application in order to optimize the efficacy of Picrotoxin for managing red palm weevil infestations.

## Figures and Tables

**Table 1:** Susceptibility of red palm weevils to different sesquiterpenes.

Treatment	LD_50_ (95% CI) (ppm)	χ2	Slope ± Standard Error
Farnesyl acetate	7867 (6911–8956)	1.18	2.45 ± 0.29
Farnesol	6559 (5543–7761)	4.09	2.07 ± 0.28
Picrotoxin	317 (538–709)	1.05	2.39 ± 0.29
β-Caryophyllene *	n/a	n/a	n/a
(+)-Cedrol *	n/a	n/a	n/a
Nerolidol *	n/a	n/a	n/a
(+)-Nootkatone *	n/a	n/a	n/a
Parthenolide *	n/a	n/a	n/a

* Failed to calculate LD_50_ values due to <50% mortality.

**Table 2:** Impact of different sesquiterpenes on the growth indices of red palm weevil larvae.

Treatments	Approximate Digestibility (AD)	ECI	ECD
Picrotoxin	74.49 ± 0.26^a^	5.07 ± 0.34^e^	6.81 ± 0.47^e^
Farnesol	62.70 ± 0.39^b^	14.09 ± 0.28^d^	22.49 ± 0.57^d^
Farnesyl acetate	60.70 ± 0.26^c^	15.79 ± 0.51	26.01 ± 0.85^c^
β-Caryophyllene	53.85 ± 0.28^de^	18.68 ± 0.15^ab^	34.70 ± 0.46^b^
(+)-Cedrol	55.13 ± 0.75^d^	18.40 ± 0.48^ab^	33.44 ± 1.32^b^
Nerolidol	53.85 ± 0.28^de^	18.61 ± 0.15^ab^	34.57 ± 0.46^b^
(+)-Nootkatone	63.89 ± 0.50^b^	17.83 ± 0.54^b^	27.95 ± 1.06^c^
Parthenolide	52.82 ± 0.88^ef^	18.75 ± 0.48^ab^	35.60 ± 1.50^ab^
Control	51.55 ± 0.23^f^	19.22 ± 0.16^a^	37.29 ± 0.48^a^

ECI stands for efficacy of conversion of ingested food; ECD stands for efficacy of conversion of digested food.

**Table 3:** Relative fold change in the expression patterns of *Rhynchophorus ferruginous* detoxification genes in the mid-gut of eighth-instar larvae using quantitative real-time PCR (qRT-PCR).

Treatments	*Cytochrome P450*	*GST*	*Esterase*
Picrotoxin	2.01 ± 0.12^g^	1.97 ± 0.11^g^	1.09 ± 0.06^d^
Farnesol	3.96 ± 0.34^f^	4.63 ± 0.41^f^	1.16 ± 0.07^d^
Farnesyl acetate	5.58 ± 0.71^e^	7.62 ± 0.69^e^	1.26 ± 0.08^cd^
β-Caryophyllene	15.71 ± 0.38^c^	18.73 ± 0.36^c^	2.54 ± 0.17^a^
(+)-Cedrol	16.26 ± 0.46^c^	19.20 ± 0.38^c^	2.37 ± 0.08^ab^
Nerolidol	17.87 ± 0.47^b^	21.96 ± 0.55^b^	2.18 ± 0.06^b^
(+)-Nootkatone	10.53 ± 0.65^d^	15.78 ± 0.87^d^	1.51 ± 0.05^c^
Parthenolide	21.41 ± 0.69^a^	25.25 ± 0.49^a^	2.20 ± 0.07^b^

*GST* stands for *Glutathione S transferase*.
